# Screening for depressive illness in adult populations

**DOI:** 10.1192/bjo.2023.560

**Published:** 2023-08-22

**Authors:** Anne M. Doherty

**Affiliations:** Department of Psychiatry, School of Medicine, University College Dublin, Ireland; and Department of Liaison Psychiatry, Mater Misericordiae University Hospital, Dublin, Ireland.

**Keywords:** Depressive episode, comorbidity, epidemiology, rating scales, primary care

## Abstract

Depression is a major cause of disability worldwide. Screening in at-risk populations is important in identifying those at most need of treatment. Pengpid et al report on high rates of incident and persistent symptoms of depression identified in an epidemiological study in a Thai population and their association with physical comorbidities. However, there are limitations to screening, due to both resource implications and the risk of diagnostic overshadowing. Although screening is useful in providing an overview of the prevalence of depressive symptoms from an epidemiological perspective, there may be justified concerns in translating this approach to clinical settings. This is especially true where the resources to provide further comprehensive assessment and treatment may be inadequate. Clinically there is a need to consider a more complete approach to screening that utilises screening tools embedded in a wider diagnostic approach which allows the detection and management of other confounding conditions.

Pengpid et al report high rates of depressive symptoms in a population aged 45 years and over living in Thailand.^1^ Analysing data from a longitudinal epidemiological study (Health, Aging, and Retirement in Thailand (HART), conducted over two time periods in 2015 and 2017) they found that nearly 10% of participants developed new depressive symptoms in the 2 year period and that 18% of those with depressive symptoms in 2015 had persistent symptoms in 2017. Examining the association between physical health conditions and depression at the two time points, they reported that diabetes, musculoskeletal conditions and a high burden of comorbidity (three or more chronic conditions) were associated with new depressive symptoms. Cardiovascular disease and a high burden of comorbidity (three or more chronic conditions) were associated with persistent depressive symptoms.

## The role of screening for depressive illnesses

Many papers in the literature have established the incidence of depressive symptoms in a range of populations and conditions. It has been well established that people with physical health conditions – diabetes, for example – have higher rates of depression.^2^ There is less clarity regarding the proportion of those studies that are based on gold-standard diagnosis and the proportion based solely on screening instruments. Graham et al, in their systematic review and meta-analysis examining the association between depression and type 2 diabetes, found 25 papers – of these, 16 studies made the ‘diagnosis’ by use of a screening tool and only 1 used a standardised semi-structured interview.^3^ This suggests that much of the literature is based on screening tools rather than on either clinical or gold-standard assessments; i.e. it refers to depressive symptoms rather than depressive episodes *per se*.

Diagnostic overshadowing is a phenomenon where symptoms common to a number of conditions may be attributed to one condition with inadequate consideration of differential diagnoses or of other factors that may be contributing to the symptoms to varying degrees. Screening instruments are not designed to distinguish between depression and other common psychological conditions, such as grief and adjustment disorder.

In addition to the overshadowing of other mental health problems, there are physical factors that may contribute to these symptoms. Putranto et al reported a significant improvement in mood following vitamin D supplementation in people with depressive symptoms and diabetes, which suggests that in this population at least the priority should be in considering the other clinical factors that might contribute to the development of depressive symptoms.^4^ It is unlikely that even the most well-designed psychological interventions will be effective in managing symptoms that are due to vitamin D deficiency, hypothyroidism, the depressant effects of alcohol, etc. Another example is poorly controlled diabetes, where a euthymic person might be expected to have difficulties with sleep, appetite, energy and concentration related to hyperglycaemia. If we screen for depression without considering the other conditions that can cause or contribute to depressive symptoms (in a pathway that does not consider differentials) such presentations may falsely inflate the prevalence of depression and may deflect clinical attention away from the person's problem.

## So is there any merit in screening?

Epidemiological studies have a huge value in informing the scientific community and policymakers about the burden of illness in a given population. Where a screening tool rather than a diagnostic instrument is used, it might be seen as a proxy measure for depression, while appreciating that not all people who screen positive would meet the criteria for a depressive episode. It would be reasonable to anticipate higher proportions in a population who have recently experienced war or famine compared with a population of people not exposed to such stressors.

The question is whether the utilisation of screening tools for depression should be the preserve of epidemiology or whether it has a role in clinical practice. One systematic review that examined screening for depression in cardiovascular care concluded that there was no benefit in terms of clinical outcomes in screening for depressive symptoms in people who have cardiovascular disease.^5^ This indicates that in a clinical setting, there is a very urgent need for a more nuanced approach to screening, more akin to a diagnostic process, where both psychological and physiological factors that may contribute to symptoms are considered.

On the other hand, when we screen for hypertension, we do not necessarily consider the cause of this to be essential to its diagnosis and treatment, and dismiss all hypertensions that are not primary hypertension. Perhaps we need a more inclusive approach to assessing depressive syndromes?

Screening instruments are easy to use. It is a more complex undertaking to provide a robust pathway and resources to manage the information acquired through screening. There may be ethical ramifications to identifying a condition in a clinical setting with inadequate resources to adequately treat.

Pengpid et al have helped to develop the evidence base regarding the comorbidities associated with depressed mood in those aged 45 years and older. It is important for clinicians to be aware of the elevated rates of depressive symptoms among people with diabetes, musculoskeletal conditions, cardiovascular disease and especially multimorbidity. Translating this epidemiology into ethical clinical practice will require further naturalistic work.

## Data Availability

Data availability is not applicable to this article as no new data were created or analysed in this work.
